# Are *Escherichia coli* Pathotypes Still Relevant in the Era of Whole-Genome Sequencing?

**DOI:** 10.3389/fcimb.2016.00141

**Published:** 2016-11-18

**Authors:** Roy M. Robins-Browne, Kathryn E. Holt, Danielle J. Ingle, Dianna M. Hocking, Ji Yang, Marija Tauschek

**Affiliations:** ^1^Department of Microbiology and Immunology, Peter Doherty Institute for Infection and Immunity, The University of MelbourneParkville, VIC, Australia; ^2^Murdoch Childrens Research Institute, Royal Children's HospitalParkville, VIC, Australia; ^3^Centre for Systems Genomics, The University of MelbourneParkville, VIC, Australia; ^4^Department of Biochemistry and Molecular Biology, Bio21 Molecular Science and Biotechnology Institute, The University of MelbourneParkville, VIC, Australia

**Keywords:** *E. coli*, diarrhoea, bacterial typing, pathotype, pathogenesis, sequence type, whole genome sequence

## Abstract

The empirical and pragmatic nature of diagnostic microbiology has given rise to several different schemes to subtype *E*.coli, including biotyping, serotyping, and pathotyping. These schemes have proved invaluable in identifying and tracking outbreaks, and for prognostication in individual cases of infection, but they are imprecise and potentially misleading due to the malleability and continuous evolution of *E. coli*. Whole genome sequencing can be used to accurately determine *E. coli* subtypes that are based on allelic variation or differences in gene content, such as serotyping and pathotyping. Whole genome sequencing also provides information about single nucleotide polymorphisms in the core genome of *E. coli*, which form the basis of sequence typing, and is more reliable than other systems for tracking the evolution and spread of individual strains. A typing scheme for *E. coli* based on genome sequences that includes elements of both the core and accessory genomes, should reduce typing anomalies and promote understanding of how different varieties of *E. coli* spread and cause disease. Such a scheme could also define pathotypes more precisely than current methods.

*Escherichia coli* is the most comprehensively studied bacterium on earth. Because it is relatively easy to manipulate genetically, it has become a popular laboratory workhorse. Its natural habitat, however, is the intestinal tract of humans and other mammals. For this reason it is used in public health as an indicator of faecal contamination of water and other consumables.

Despite its ubiquity as a commensal, *E. coli* is also an important pathogen of humans and domestic animals. It can become established and cause disease in tissues other than the intestinal tract. These so-called extraintestinal pathogenic *E. coli* (ExPEC) are important causes of wound infection, urinary tract infection, peritonitis, pneumonia, meningitis, and septicaemia. The ExPEC group includes named subtypes, such as uropathogenic *E. coli* (UPEC), neonatal meningitis-associated *E. coli* (NMEC), and sepsis-associated *E. coli* (SEPEC) (Pitout, 2012; Leimbach et al., 2013).

Infections caused by ExPEC are usually opportunistic, i.e., they occur most often in hosts who are compromised in some way, such as by having a dysfunctional urinary tract or systemic immunocompromise due to neutropenia or extremes of age. Nevertheless, some ExPEC strains are better equipped to cause extraintestinal infections than others due to factors that facilitate their ability to colonise tissues. These include type I fimbriae, pyelonephritis-associated pili (PAP), and AfA/Dr adhesins in the case of UPEC, or the K1 polysaccharide capsule, which allows NMEC and SEPEC to evade complement-mediated killing (Pitout, 2012; Leimbach et al., 2013).

The pathotypes of *E. coli* that are associated with intestinal disease are known collectively as intestinal pathogenic *E. coli* (IPEC) or diarrheagenic *E. coli* (DEC)—although not all of the subtypes in this group necessarily cause diarrhoea. The individual pathotypes of DEC include enteropathogenic *E. coli* (EPEC), enteroinvasive *E. coli* (EIEC), enterotoxigenic *E. coli* (ETEC), enterohemorrhagic *E. coli* (EHEC), enteroaggregative *E. coli* (EAEC), diffusely-adherent *E. coli* (DAEC), and adherent-invasive *E. coli* (AIEC) (Nataro and Kaper, 1998; Kaper et al., 2004; Croxen et al., 2013). In addition, the entire genus *Shigella* is a DEC pathotype, which closely resembles EIEC in terms of virulence attributes and pathogenicity, but is distinguishable from other strains of *E. coli* by virtue of its biochemical activity (Lan et al., 2004). Accordingly, shigellae can be regarded as members of the EIEC pathotype.

Each DEC pathotype represents a collection of strains that possess similar virulence factors to each other and cause similar diseases with similar pathology. Unlike ExPEC, where there are no specific virulence determinants that exclusively define each subtype, most DEC pathotypes are defined by the possession of one or more pathotype-specific virulence markers, and sometimes by the absence of others. Several of the defining markers of DEC pathotypes are proven virulence determinants of that pathotype, but for EAEC, DAEC, and AIEC the role of these markers in virulence is not proven (Table 1).

**Table 1:** **Virulence-associated markers of diarrheagenic ***E. coli*** from humans**.

**Pathotype**	**Defining marker**	**Essential virulence determinant(s)**	**Location of essential virulence determinant(s)**	**Major diagnostic target(s) for PCR**	**Other diagnostic target(s)**
EPEC	LEE PAI	LEE PAI	Pathogenicity island	*eae*	*bfpA*^a^
EIEC/Shigella	pINV	pINV	Plasmid	*ipaH*	Other *ipa* genes
ETEC	ST or LT	ST and/or LT plus colonisation factors	Plasmid; transposon	*elt, est*	
EHEC	Shiga toxin	Shiga toxin 1 and/or 2	Prophages	*stx1, stx2*	*eae*^a^, *ehxA*^a^
EAEC	pAA; aggregative adhesion	Not known	Plasmid (probably); possibly others	*aggR, aatA, aaiC*	
DAEC	Afa/Dr adhesins	Not known	Not known	Afa/Dr adhesins^b^	
AIEC	Adherent-invasive phenotype	Not known	Not known	none	none

*aaiC, gene for a secreted protein of enteroaggregative E. coli; aatA, gene for a transporter protein of enteroaggregative E. coli; Afa, afimbrial adhesin; aggR, gene for a transcriptional regulator; AIEC, adherent-invasive E. coli; bfpA, gene for a structural protein of bundle-forming pili; DAEC, diffusely-adherent E. coli; EAEC, enteroaggregative E. coli; EIEC, enteroinvasive E. coli; elt, gene for heat-labile enterotoxin; EPEC, enteropathogenic E. coli; est, gene for heat-stable enterotoxin; ETEC, enterotoxigenic E. coli; ipaH, gene for a type 3-secreted effector protein of enteroinvasive E. coli and Shigella; LEE PAI, locus of enterocyte effacement pathogenicity island; LT, heat-labile enterotoxin; pAA, virulence plasmid of enteroaggregative E. coli; pINV, virulence plasmid of enteroinvasive E. coli and Shigella; ST, heat-stable enterotoxin*.

a *Not present in all strains*.

b *These are under review following concerns about specificity*.

## DEC pathotypes

In this section, we provide a brief overview of DEC pathotypes with an emphasis on their defining characteristics and key virulence determinants (where known). We also point out some important gaps in the understanding of certain pathotypes. For more detailed information on DEC in general, readers are referred to reviews by Nataro and Kaper (1998), Kaper et al. (2004), Clements et al. (2012), and Croxen et al. (2013). In the bibliography, we have also included references to review articles dealing with individual DEC pathotypes.

### Enteropathogenic *E. coli* (EPEC)

EPEC was the first pathotype of DEC to be discovered, and is an important cause of diarrhoea and premature death in children, especially in developing countries (Robins-Browne, 1987). As a group, EPEC is characterised by the presence of the locus of enterocyte effacement (LEE) pathogenicity island (McDaniel and Kaper, 1997; Robins-Browne and Hartland, 2002; Croxen et al., 2013). This ~40-kbp island encodes (i) an outer membrane adhesive protein, known as intimin that is encoded by the *eae* gene, (ii) a type 3 protein secretory system, (iii) several type 3-secreted effectors, including the Tir protein which is the translocated receptor for intimin (Kenny et al., 1997).

Expression of the LEE is associated with distinctive attaching-effacing lesions in the intestinal epithelium which characterise EPEC pathology (Moon et al., 1983; Tzipori et al., 1985). Almost all genes within the LEE are required for the production of these lesions, and studies in adult volunteers have demonstrated that intimin and EspB, a key component of the type 3 secretion system, are essential virulence determinants of EPEC (Donnenberg et al., 1993; Tacket et al., 2000). An accessory virulence determinant, which EPEC also requires for virulence in humans, is the bundle-forming pilus (BFP) (Girón et al., 1991; Bieber et al., 1998). Some human isolates of EPEC naturally lack BFP, but may cause disease (Trabulsi et al., 2002; Nguyen et al., 2006). These strains, known as atypical EPEC are associated with persistent diarrhoea in children (Nguyen et al., 2006). Atypical EPEC are genetically diverse and appear to vary in virulence (Tennant et al., 2009; Ingle et al., 2016a).

### Enterohemorrhagic *E. coli* (EHEC)

EHEC first came to attention as the cause of two outbreaks of haemorrhagic colitis (HC) in the USA during 1982 (Riley et al., 1983). The defining virulence determinant of EHEC is the phage-encoded Shiga toxin (also known as Verotoxin), of which there are several varieties (O'Loughlin and Robins-Browne, 2001; Melton-Celsa et al., 2012). Although volunteer studies with EHEC are prohibited for ethical reasons, vast quantities of epidemiological data leave no doubt that Shiga toxin is responsible for the life-threatening manifestations of EHEC infections, namely, HC and the haemolytic uraemic syndrome (HUS). Evidence supporting a role for Shiga toxin in these conditions include the observation that infections with other bacteria which produce Shiga toxin, such as *Shigella dysenteriae* serotype 1 and occasional strains of EAEC, may also cause HC and HUS (Rohde et al., 2011; Walker et al., 2012).

Not all strains of Shiga toxin-producing *E. coli* (STEC or VTEC) cause HC or HUS, and the term “EHEC” is generally reserved for those that do. Thus, although all EHEC are STEC, not all STEC are EHEC. The properties that distinguish EHEC from those STEC that do not cause HC or HUS are accessory virulence factors which allow the bacteria to adhere to the intestinal epithelium, such as the LEE pathogenicity island in so-called “typical EHEC” or a number of other adhesins that are present in LEE-positive and/or LEE-negative strains (reviewed in McWilliams and Torres, 2014).

Typical EHEC strains of serotype O157:H7 also generally carry a virulence-associated plasmid, known as pO157, which encodes a number of putative virulence determinants (Burland et al., 1998). Related plasmids occur in EHEC of other serogroups, including O26, O103, O111, and O145 (Ogura et al., 2009). One of the virulence-associated factors encoded by these plasmids is a serum-sensitive haemolysin, known as EHEC haemolysin or enterohaemolysin. Many EHEC isolates produce this protein, including some that carry plasmids only distantly related to pO157 (Beutin et al., 1989). Accordingly, the production of enterohaemolysin can be used as a diagnostic marker of EHEC (Feldsine et al., 2016). Interestingly, enterohaemolysin is also produced by some LEE-positive, Shiga toxin-negative strains of *E. coli* obtained from cattle and sheep (Cookson et al., 2007). This observation provides evidence of the evolutionary relationship between atypical EPEC and EHEC, which is also evident from the high degree of relatedness between atypical EPEC strains of serotype O55:H7 and EHEC O157:H7 (Feng et al., 1998).

### Enterotoxigenic *E. coli* (ETEC)

ETEC is a leading cause of diarrhoea in children in developing countries and in travellers to these countries (Qadri et al., 2005; Lanata et al., 2013). ETEC is also an important cause of diarrhoea in domestic animals, notably calves and piglets, where ETEC-induced diarrhoea is of considerable economic importance (Nagy and Fekete, 1999; Fairbrother et al., 2005).

As the name suggests, the ETEC pathotype is defined by the capacity of the bacteria to produce one or more enterotoxins. In ETEC the specific enterotoxins are the heat-labile and heat-stable enterotoxins (LT and ST) and their various subtypes (Qadri et al., 2005). The two major subtypes of ST are STa (also known as STI) and Stb (STII), of which only STa is important in humans (Qadri et al., 2005; Taxt et al., 2010). Most ETEC strains isolated from humans with diarrhoea produce STa, often together with LT. The role of each of these toxins in disease has been established in volunteer studies (Levine et al., 1977, 1979).

Both ST and LT exert their maximum impact on water and electrolyte transport in the small intestine. In order to deliver these toxins to the small intestinal epithelium, ETEC need to attach to epithelial cells, which they achieve by means of specific colonisation factors (Qadri et al., 2005; Madhavan and Sakellaris, 2015). These factors are highly variable structurally and antigenically, and also differ between isolates from humans and animals. In several instances, the role of colonisation factors as accessory virulence determinants has been demonstrated experimentally (Qadri et al., 2005; Madhavan and Sakellaris, 2015).

### Enteroinvasive *E. coli* (EIEC)

EIEC are closely related to *Shigella*, especially in terms of the disease they cause, i.e., bacillary dysentery, and their key virulence determinant: a plasmid known as pINV. This plasmid encodes a type 3 secretion system and a number of effectors that allow shigellae/EIEC to penetrate epithelial cells, move within these cells and invade neighbouring cells (Marteyn et al., 2012). Both shigellae and EIEC carry several other putative virulence determinants including adhesins and secreted toxins, but pINV, which appears to be restricted to these bacteria, is the key to their virulence (Marteyn et al., 2012; Croxen et al., 2013).

EIEC and shigellae exemplify the changes that *E. coli* can make to adjust to a pathogenic lifestyle (Day et al., 2001). Thus, by acquiring pINV, and other genetic elements that allow the bacteria to adopt an intracellular lifestyle, the capacity of *E. coli* to live inside cells is continuously enhanced by the deletion or inactivation of genes that are inimical to this lifestyle (Day et al., 2001; Feng et al., 2011; Prosseda et al., 2012). Examples of such genes include some that encode anti-virulence factors, such as *nadA, nadB*, and *ompT*, and those for metabolic pathways such as lysine decarboxylation, the end products of which restrict intracellular growth (Day et al., 2001; Prunier et al., 2007). Moreover, since flagella are not required for colonisation of the large intestine or for motility within cells, all shigellae and many strains of EIEC are non-motile. The capacity of *E. coli* to adapt to new environments in this way provides fascinating insights into the extraordinary versatility of this species as a pathogen.

### Enteroaggregative *E. coli* (EAEC)

**Figure 1:**
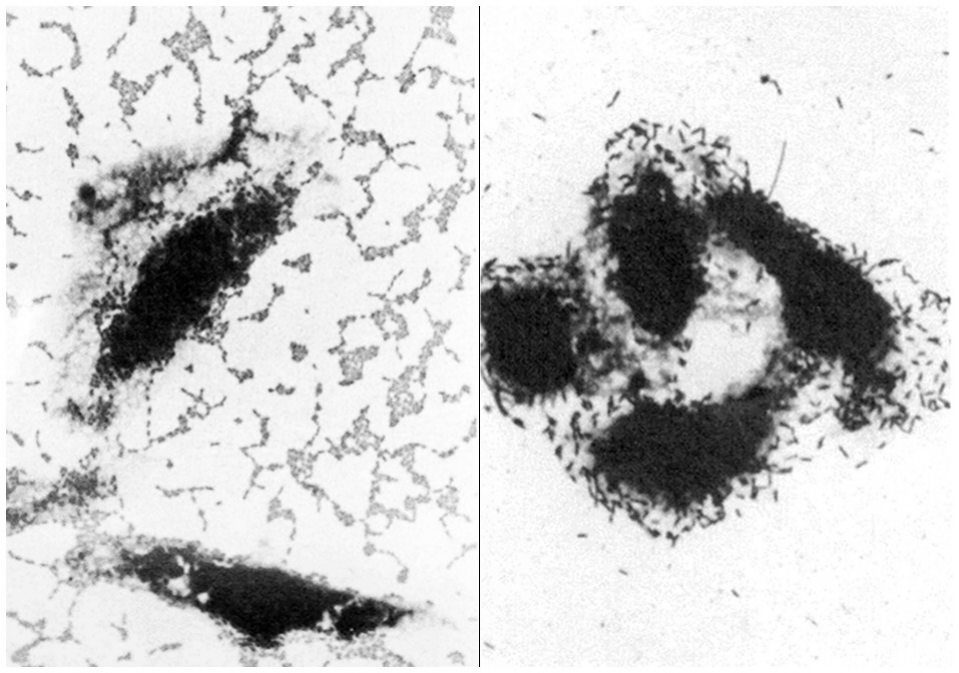
**Light micrographs showing the distinctive patterns of adherence of enteroaggregative ***E. coli*** (left) and diffusely-adherent ***E. coli*** (right) to cultured epithelial cells (adapted from Nataro et al., 1987)**. These patterns were responsible for the names of these pathotypes and were originally used to identify them *in vitro*.

This relatively recently discovered *E. coli* pathotype is mainly associated with paediatric diarrhoea in developing countries, but has also been linked to diarrhoea in adults, including travellers (Okeke and Nataro, 2001; Harrington et al., 2006). EAEC was originally identified by its characteristic “stacked-brick” pattern of adherence to tissue culture cells *in vitro* (Nataro et al., 1987; Figure 1). This phenotype is attributable to one of several different hydrophobic aggregative fimbriae, known as AAF/I, AAF/II, AAF/III, and AAF/IV, encoded by pAA or similar plasmids. Other putative virulence factors of EAEC include (i) a pAA-encoded cytotoxin (Pet), (ii) a pAA-encoded heat-stable enterotoxin, known as enteroaggregative stable toxin (EAST-1) that is related to STa of ETEC, but not restricted to EAEC, and (iii) ShET1, a putative enterotoxin that is also found in *Shigella flexneri* (Okeke and Nataro, 2001; Croxen et al., 2013). Although the pathogenicity of EAEC is evident from foodborne outbreaks in several countries and infection studies of volunteers (Nataro et al., 1995; Harrington et al., 2006), the contribution of these and other putative virulence-associated determinants of EAEC is not known (Harrington et al., 2006; Croxen et al., 2013). As with atypical EPEC, EAEC are genetically diverse with the likelihood that some types are more virulent than others (Boisen et al., 2012; Zhang et al., 2016).

In 2011, a Shiga toxin-producing derivative of an EAEC strain of serotype O104:H4, shot to prominence by causing a major foodborne outbreak of diarrhoea and HUS in Germany, with serious outcomes for human health and the international food trade (Buchholz et al., 2011; Rohde et al., 2011).

Few studies of diarrhoea today use the aggregative adherence phenotype to identify EAEC. Instead most investigators target the pAA-borne genes, *aatA* and *aggR* (that encode a transporter of a virulence protein and a virulence regulator, respectively), or the chromosomally-encoded *aaiC* gene which is also associated with virulence (Table 1; Panchalingam et al., 2012). Although PCR-based identification of EAEC is convenient, the presence or absence of these genes does not necessarily concur with the aggregative phenotype, nor is it known whether this phenotype or the presence of *aatA, aggR* and/or *aaiC* is the more reliable predictor of virulence (Weintraub, 2007; Croxen et al., 2013). This issue is not trivial, because until it is resolved we will lack a clear definition of what really constitutes EAEC.

### Diffusely-adherent *E. coli* (DAEC)

As with EAEC, DAEC were originally identified by their distinctive pattern of adherence to tissue culture cells (Scaletsky et al., 1984; Nataro et al., 1985, 1987; Figure 1). The first determinant of diffuse adherence to be identified was an autotransporter protein, known as AIDA-I, for the Adhesin Involved in Diffuse Adherence (Benz and Schmidt, 1992). *E. coli* strains that express AIDA-1, however, generally carry other virulence determinants, such as STb, making them ETEC (Dubreuil, 2010), or the LEE pathogenicity island, making them EPEC (Servin, 2005, 2014; Table 1). Accordingly, few of these strains are considered DAEC, despite their phenotype.

AIDA-I-negative DAEC strains typcially express Afa/Dr adhesins and cause urinary tract infections, placing them in the UPEC subgroup of ExPEC. Although *E. coli* that express afimbrial adhesins (Afa) and Dr fimbriae have been associated with diarrhoea in children, the specificity of the probes and PCR primers that were used to detect and identify these bacteria is questionable, in that they may also react with EAEC and some other types of *E. coli* (Servin, 2014). This, and the fact that two prototypical DAEC strains failed to cause diarrhoea in volunteers who ingested up to 10^10^ colony-forming units, casts doubt on the role of DAEC in diarrhoea, notwithstanding considerable evidence of the deleterious effects of these bacteria on intestinal epithelial cells *in vitro* (reviewed in Servin, 2014).

### Adherent-invasive *E. coli* (AIEC)

AIEC are unusual amongst DEC pathotypes in that they are not associated with diarrhoea. Instead they are thought to contribute to the development of Crohn's disease, which is a chronic inflammatory bowel disease. The aetiology of Crohn's disease is uncertain, but is likely to involve both host and environmental factors (Alhagamhmad et al., 2016). AIEC strains are discernible from other varieties of *E. coli*, including commensals, by virtue of their ability to adhere to and invade epithelial cells and to replicate within macrophages (Martinez-Medina et al., 2009). Analysis of whole genome sequences of several AIEC isolates, however, has shown that the AIEC phenotype may not be due to one or more specific virulence determinants (O'Brien et al., 2016), suggesting that the distinctive phenotype of these bacteria may result from metabolic processes that enhances growth in tissues affected by Crohn's disease. Thus, although AIEC are recovered more commonly from patients with Crohn's disease than from healthy people, it is unclear whether these bacteria contribute to the pathogenesis of Crohn's disease or are merely adapted to or enriched in intestinal tissue affected by this disease.

## *E. coli* genomics

The first complete genome sequence of an *E. coli* strain (*E. coli* K-12) was published in 1997 (Blattner et al., 1997). Since then many thousands of *E. coli* isolates from a wide range of sources have also been sequenced, although most of these genomes have not been fully assembled into a finished and complete genome sequence. Nevertheless, from the available data we can glean that the size of the *E. coli* genome (which includes plasmids and prophage) ranges from approximately 4.6 million base pairs (Mbp) to around 5.9 Mbp—a difference of more than 1.3 Mbp.

Each individual *E. coli* strain carries between 4200 and 5500 genes. As more *E. coli* strains are sequenced the core genome (i.e., the backbone of chromosomal genes that are present in every *E. coli* strain) shrinks. The size of the core genome currently stands at fewer than 1500 genes and will to continue to diminish, albeit slowly, as more strains are sequenced. Genes that are not part of the core are referred to as the accessory genome. These include all of the genes that encode bacteriophage elements, virulence determinants and acquired resistance to antimicrobials. The *E. coli* pangenome—the total number of unique genes that have been identified in *E. coli—*comprises more than 22,000 and will continue to increase as more strains are sequenced.

All of the genes for *E. coli* virulence determinants were most likely acquired by horizontal gene transfer from other bacteria via plasmids, bacteriophages, pathogenicity islands, and transposons (Leimbach et al., 2013; Table 1). Thus, every *E. coli* strain comprises a mosaic of core and accessory genes, with almost all of the latter, including the virulence determinants of DEC, being transmissible between strains. For these reasons, it is inevitable that new pathotypes of DEC will continue to emerge, either through novel assemblies of *E. coli* virulence determinants, as in the case of EHEC (Feng et al., 1998) and Shiga toxin-producing EAEC (Rohde et al., 2011), or through the acquisition of virulence genes from other bacterial species.

## *E. coli* subtypes

Apart from pathotype, individual strains of *E. coli* can be subtyped using a variety of criteria that may vary between individual strains. These include sequence type, serotype, pulsotype, phage type, and biotype.

### Sequence type

The conserved nature of the *E. coli* core genome allows determination of the genetic distance between strains based on nucleotide polymorphisms in shared genes. For more than a decade multi-locus sequence typing (MLST), in which sequence types (STs) are defined on the basis of combinations of allelic variation in 6–11 so-called “housekeeping genes” (Maiden et al., 1998), has been the gold standard for DNA sequence-based typing of bacterial pathogens. Three MLST schemes have been proposed for *E. coli*, each based on a different set of 7–8 genes (Reid et al., 2000; Wirth et al., 2006; Jaureguy et al., 2008), of which the 7-locus scheme of Mark Achtman appears to be the most stable and congruent with whole genome phylogenies (Chaudhuri and Henderson, 2012; Clermont et al., 2015). The principle of MLST has recently been extended to core gene MLST (cgMLST) (Maiden et al., 2013), and a new *E. coli* scheme incorporating more than 2500 genes is now available (alongside the 7-locus scheme of Mark Achtman) in the Enterobase database hosted at the Warwick Medical School (http://enterobase.warwick.ac.uk). Sequence typing has proved useful in many settings, e.g., in tracing the spread of particular strains in different regions, such as *E. coli* ST131, a multidrug resistant UPEC clone (Nicolas-Chanoine et al., 2014; Petty et al., 2014).

### Serotype

Serotyping based on antigenic variation in the surface O- (polysaccharide) and H- (flagella) antigens of *E. coli* was previously used for the preliminary identification of DEC pathotypes. Indeed, much of the early evidence linking EPEC to the cause of outbreaks of diarrhoea was based on the antigenic relatedness of strains obtained from patients in different locations (Robins-Browne, 1987). ETEC, EIEC, EHEC, and EAEC also belong to a limited number of serotypes, but serotyping is no longer used for the preliminary identification of these categories, having been replaced by direct testing for the presence of virulence-associated genes (Table 1). Moreover, *E. coli* serotypes are not immutable, and can change due to mutation or phage-mediated transduction (Mavris et al., 1997; Kido and Kobayashi, 2000). The superiority of sequence typing over serotyping is illustrated by the ST131 UPEC pandemic strain, in which most isolates are serotype O25b:H4, but some are serotype O16:H5 (Nicolas-Chanoine et al., 2014). Importantly, *E. coli* serotypes can be reliably predicted from whole genome sequences (Ingle et al., 2016b). Indeed, *in-silico* serotyping offers a number of advantages over traditional serotyping, including the non-reliance on typing sera that may vary in quality, and the ability to type strains that do not express the O- or H-antigens *in vitro* or that autoagglutinate (Ingle et al., 2016b). For these reasons, *in-silico* serotyping is likely to replace traditional serotyping in future.

Nevertheless, many food microbiology laboratories currently use serotyping for the preliminary identification of EHEC, most notably *E. coli* O157:H7 and the so-called “big six” serogroups (O26, O45, O103, O111, O121, and O145) of EHEC strains (Brooks et al., 2005).

Interestingly, even the identification of serotype, together with the demonstration of a suite of shared virulence genes, may not provide sufficiently refined information to identify a particular subclone or clade of EHEC (Manning et al., 2008). In such instances, further subtyping may be required to track outbreaks. Traditionally, this has included phage typing (which is based on the susceptibility of isolates to infection with one or more specific virulent bacteriophages) or typing based on restriction fragment length polymorphism (pulsotyping), which permits the discernment of outbreak strains from background “noise” (Bender et al., 1997). The value of pulsotyping is exemplified by PulseNet, a surveillance network of public health laboratories that use DNA fingerprinting for the early identification of common sources of foodborne outbreaks of disease (Swaminathan et al., 2001). More recently, public health laboratories have been shifting to analysis of whole genome single nucleotide polymorphisms (SNPs) to trace outbreaks of *E. coli* and other foodborne pathogens. This approach first captured the attention of the international public health community during the high-profile 2011 outbreak of diarrhoea and HUS in Germany caused by Shiga toxin producing EAEC (Buchholz et al., 2011; Rohde et al., 2011), and is now being used for routine analysis in many laboratories, e.g., to investigate *E. coli* O157:H7 outbreaks by Public Health England (Cowley et al., 2016), and the GenomeTrakr project established by the US Food and Drug Administration (Allard et al., 2016).

### Biotype

Biotyping was once relied upon to group and separate individual strains of *E. coli*, particularly in the period before serotyping became established for this purpose. Currently, biotyping is still used to distinguish shigellae from other varieties of *E. coli*. Although at present there is no comprehensive scheme to predict *E. coli* biotype from whole genome sequences, this may be possible in future should biotyping still be required.

Biochemical profiles also play a central role in the isolation and preliminary identification of *E. coli* strains in general, on media such as McConkey and eosin methylene blue agar, and of EHEC on sorbitol MaConkey (SMAC) agar and CHROMagar STEC medium (de Boer et al., 2015).

### Pathotype

As mentioned above, the subdivision of DEC into pathotypes has may uses. However, some isolates do not comply with the standard pathotyping scheme (Table 2). Such strains include isolates of EPEC that carry genes for the heat-labile enterotoxin of ETEC (Dutta et al., 2015); and strains of ETEC and EAEC that secrete Shiga toxin (Zhang et al., 2007; Buchholz et al., 2011). Even *Shigella dysenteriae* type 1, which carries the Shiga toxin gene on its chromosome is atypical, as far as the *Shigella* biotype is concerned, since no other strain in this “genus” produces this toxin. In addition, *Shigella boydii* serotype 13 is unusual in that it carries the LEE pathogenicity island of EPEC (Walters et al., 2012), although this particular clone is evidently incorrectly classified, being more closely related to *E. albertii* than to *E. coli* (Hyma et al., 2005). *E. albertii* is a disctinct *Escherichia* species that is characterised in part by its carriage of the LEE pathogenicity island (Huys et al., 2003).

**Table 2:** **Examples of clinically significant diarrheagenic ***E. coli*** strains that do not comply with established pathotypes**.

**Strain**	**Comments**	**References**
Shiga-toxin producing EAEC	Some investigators have deemed these to a new pathotype named STEAEC	Clements et al., 2012
Shiga-toxin producing ETEC	Most of these strains are associated with pig edema disease	Zhang et al., 2007
LT-producing EPEC	We have found this uncommon hybrid in our studies of paediatric diarrhoea (unpublished)	Dutta et al., 2015
*Shigella* B13 carrying the LEE pathogenicity island	This clone is more closely related to *E. albertii* than to *E. coli*	Hyma et al., 2005; Walters et al., 2012

*EAEC, enteroaggregative E. coli; EPEC, enteropathogenic E. coli; ETEC, enterotoxigenic E. coli; LEE, locus of enterocyte effacement; LT, heat-labile enterotoxin; STEAEC, Shiga-toxin producing enteroaggregative E. coli*.

Hybrid strains of *E. coli* pathotypes are not surprising given the mobility of most of the genes that encode virulence in DEC. What is perhaps more surprising is that hybrids don't occur more often. In this regard, DEC strains that infect humans seem somewhat limited in the combinations of virulence determinants that occur together, other than those that are already well characterised (Table 1). Thus, whereas EPEC, EHEC, ETEC, and shigellae have all emerged on several different occasions (Pupo et al., 2000; Sahl et al., 2011; von Mentzer et al., 2014; Ingle et al., 2016a), hybrids of these are uncommon (Nyholm et al., 2015). By contrast, some EPEC strains from animals carry colonisation fimbriae that closely resemble those from ETEC (Adams et al., 1997), and ETEC from swine may express Shiga toxin as well as STa and/or STb (Zhang et al., 2007; DebRoy et al., 2010).

A particular limitation of pathotyping concerns its limited capacity to accommodate new strains that do not comply with known categories. These include Shiga toxin producing strains of EAEC, which some authors have assigned to a new pathotype, designated Shiga-toxin producing enteroaggregative *E. coli* (Clements et al., 2012). This is not unreasonable considering that EHEC, which is a well-accepted pathotype itself, appears to have emerged relatively recently from EPEC (Feng et al., 1998), but the nomenclature is unwieldy and inflexible. For example, it may be more accurate to use the term “Shiga toxin producing atypical EPEC” for EHEC given the origin of these strains, and Shiga toxin producing ETEC for the bacteria that cause oedema disease in pigs.

Another problem with the current definitions of DEC pathotypes is that some strains are defined in part by negative criteria. For example, EPEC is defined as having the LEE pathogenicity island, but lacking Shiga toxin (otherwise it would be EHEC), and atypical EPEC is defined as lacking both Shiga toxin and bundle-forming pili (Kaper, 1996; Trabulsi et al., 2002). We believe that characterising pathogens on the basis of their lack of one or more virulence determinants may group several types of distantly related or unrelated bacteria together, and cause some distinct pathogenic categories with uncharacterised virulence determinants to be overlooked.

## Conclusion

The ability to divide *E. coli* into subtypes is essential to understand the epidemiology and pathogenesis of particular clones. The use of sequence typing, biotyping, serotyping, and pathotyping to group similar bacteria together while separating them from others is helpful in many circumstances, such as when tracing outbreaks, but can be misleading when serotypes change or classification systems struggle to accommodate novel strains.

The subdivision of DEC into pathotypes is critical for understanding how these bacteria cause disease. The identification of pathotypes is also invaluable clinically (to determining prognosis and guide clinical management) and epidemiologically to detect outbreaks and estimate the contribution of different types of DEC to the overall burden of disease, as well as for the control of these diseases by public health interventions and immunisation (Levine et al., 1983; Sjöling et al., 2015).

Whole genome sequencing of *E. coli* strains has vastly enhanced our understanding of the evolution and pathobiology of this highly adaptable and versatile species. A major advantage of whole genome sequencing is that most subtypes and other properties can be predicted with a high degree of accuracy from sequence data. Combined with clinical, pathological and epidemiological metadata, whole genome sequencing will also permit elucidation of which strains within a subtype are more virulence than others. For these reasons, we expect that some of the typing schemes in current use will eventually be replaced by a system that is based on a combination of genes within the core genome (probably cgMLST) and the accessory genome, comprising major virulence determinants and associated pathogenic potential. In this regard, the coordinated sharing of whole genome sequence data via GenomeTrakr, coupled with standardised extraction of *E. coli* typing information including sequence type, serotype, pathotype and antimicrobial resistance from genome data using tools such as Enterobase is likely to become the new gold standard for *E. coli* analysis. Thus, although whole genome sequencing will not replace pathotyping in the short-term, it should, together with clinical, field, and experimental data, be used to enhance our understanding of what constitutes a pathotype, while allowing for more pathotypes to be identified by permitting the identification of particular combinations of genes that are associated with specific clinical syndromes and pathology. This is particularly important for loosely defined pathotypes, such as EAEC, DAEC, AIEC, and atypical EPEC.

## Author contributions

All of the authors contributed to the preparation of the manuscript, and to the ideas and concepts contained in it.

### Conflict of interest statement

The authors declare that the research was conducted in the absence of any commercial or financial relationships that could be construed as a potential conflict of interest.
